# Analysis of Antioxidant Compounds in *Vitex negundo* Leaves Using Offline 2D-LC-ECD and LC-MS/MS

**DOI:** 10.3390/molecules29133133

**Published:** 2024-07-01

**Authors:** Qimei Wu, Jinfen Zheng, Yan Yu, Zhirong Li, Ying Li, Chengfeng Hu, Yaping Zhou, Rongxiang Chen

**Affiliations:** 1 School of Pharmacy, Zunyi Medical University, Zunyi 563000, China; 2 School of Basic Medicine, Zunyi Medical University, Zunyi 563000, China

**Keywords:** offline two-dimensional liquid chromatography, electrochemical detection, tandem mass spectrometry, antioxidant activity, *Vitex negundo* leaves

## Abstract

*Vitex negundo* has strong antioxidant activity, but its primary antioxidant components are not clear. In this study, the antioxidant components were screened by offline two-dimensional liquid chromatography coupled with electrochemical detection (2D-LC-ECD) and subsequently assessed using liquid chromatography-tandem mass spectrometry (LC-MS/MS) identification, radical scavenging capacity, and molecular docking. Various fractions were isolated from *Vitex negundo* leaves, and 39 antioxidant components were screened and identified. All of the fractions containing the antioxidant components exhibited certain antioxidant activity. Correlation analysis revealed a strong correlation between the response of LC-ECD and the in vitro antioxidant activity of the fractions. Molecular docking demonstrated that components with high response to LC-ECD exhibited robust interaction with antioxidant-related target proteins. The main antioxidant components of *Vitex negundo* leaves were isoorientin, chlorogenic acid, agnuside, cynaroside, and scutellarin. The 2D-LC-ECD combined with LC-MS/MS was rapid and effective in screening the antioxidant components in *Vitex negundo* leaves and could also provide technical support for the discovery of antioxidant components with different polarities and contents in other medicinal and edible plants.

## 1. Introduction

*Vitex negundo* L var. *cannabifolia* (Siebold et Zucc) Hand. -Mazz (*Vitex negundo*) originates from India and is mainly distributed in temperate and tropical regions [1]. Its leaves possess antioxidant, anti-inflammatory, antidiabetic, antitumor, and antimicrobial properties [2,3]. It is widely used in medicine, food, and agricultural products in China [4]. Studies have shown that the leaves of *Vitex negundo* primarily consist of flavonoids [5], terpenoids [6], phenolic acids [7], and lignans [8], and the extracts have significant antioxidant activity [9]. Although a large number of compounds have been identified, the contribution of different compounds to the antioxidant activity of *Vitex negundo* leaves is still unclear.

Previous research have identified that natural compounds such as phenolic acids, flavonoids, and other phenolic compounds sourced from medicinal plants exhibit potent antioxidant properties [10]. Compared to synthetic antioxidants, natural antioxidants have higher safety and multiple functional characteristics [11]. Spectrophotometric techniques, such as 1,1-diphenyl-2-trinitrophenylhydrazine (DPPH) free radical scavenging ability, 2,2′-hydrazine-di-3-ethylbenzothiazoline-6-sulfonic acid (ABTS) free radical scavenging ability, oxygen radical absorbance capacity (ORAC), and ferric reducing antioxidant power (FRAP), are most frequently used to assess the antioxidant activity in medicinal plants [12,13,14]. However, the effectiveness of screening antioxidants by isolating individual compounds is limited [15], and a single component cannot fully represent the biological activity of the entire plant [16]. To improve the efficiency, some studies have used HPLC-UV with online DPPH or ABTS reaction to screen antioxidant components in medicinal plants [17], but the sensitivity is relatively low. In addition, the combination of HPLC-UV/DAD fingerprint and comprehensive evaluation of antioxidant activity, as well as gray correlation analysis and partial least squares regression analysis, can be used to screen antioxidant components [18]. Nevertheless, it typically necessitates the examination of numerous samples and depends on statistical analysis, which can produce varying outcomes depending on the statistical techniques employed [19]. Therefore, it is imperative to develop a rapid, accurate, highly stable, and sensitive screening method. Electrochemical analysis techniques, including cyclic voltammetry [20], differential pulse voltammetry [21], and flow-injection amperometry [22], measure the current or charge generated by the oxidation–reduction reaction of the analyte on the working electrode surface. These techniques have higher sensitivity than spectrophotometry and are widely used in medicine [23], biomedicine [24], food [25,26], and other fields. Previous reports have shown that HPLC with electrochemical detection (ECD) is a feasible method for screening antioxidant components such as phenolic acids and flavonoids in medicinal plants and foods [27].

The complexity of components in medical plants makes it difficult to use one-dimensional liquid chromatography (1D-LC) to separate and analyze them simultaneously. However, this issue can be resolved effectively by employing two-dimensional liquid chromatography (2D-LC), which has been successfully applied to the analysis of bioactive ingredients in medicinal plants [28]. In particular, the comprehensive 2D-LC can achieve higher peak capacity and better separation and provide a comprehensive description of the chemical composition of medicinal plants. Offline 2D-LC offers greater flexibility over the online 2D-LC in terms of operation mode. On the one hand, there are no restrictions on the separation and analysis of the second-dimensional fractions, allowing for the selection of a chromatographic column with higher efficiency to better separate the fractions obtained in the first-dimensional fractions. On the other hand, the biological activity of the first-dimensional fractions can be studied separately. In addition, compared with the original sample, the simplified first-dimensional fractions can reduce matrix interference and facilitate mass spectrometry analysis, making it easier to identify compounds [29].

The mechanism of antioxidants in vivo is to enhance the antioxidant capacity of cells by inhibiting the formation of reactive oxygen species and increasing the level of antioxidant enzymes [30]. Molecular docking can preliminarily predict the binding mechanism between active components and antioxidant activity at the molecular level. It is one of the most widely used methodologies because it does not require trial and is inexpensive [31]. In this study, offline 2D-LC-ECD was employed to prepare and analyze the fractions of *Vitex negundo* leaves, evaluate their antioxidant activity in vitro, and identify them by liquid chromatography–tandem mass spectrometry (LC-MS/MS), with the objective of screening out antioxidant compounds. Finally, molecular docking technology was utilized to verify their interaction with antioxidant-related target proteins.

## 2. Results and Discussion

### 2.1. Separation of the Antioxidant Components in Vitex negundo Leaves by Offline 2D-LC-ECD

**Figure 1:**
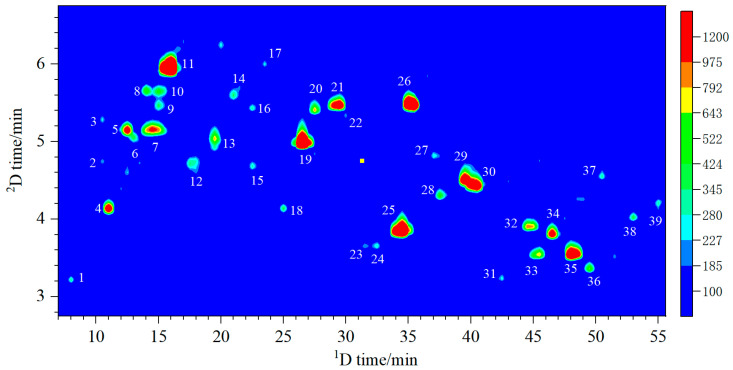
The 2D-LC-ECD chromatogram of *Vitex negundo* leaves.

In the ECD, electroactive components undergo redox reactions when passing through the electrode surface at a certain potential. The extent of the potential is crucial in determining whether the compound reacts entirely. The study explored various potentials ranging from 100 to 750 mV. The findings affirmed that the total peak area rose progressively with the increase in potential up to saturation that occurs at 700 mV. This evidence supports the decision to use 700 mV as the designated potential condition. Under the optimal conditions, LC-ECD chromatograms of different fractions were obtained, and the results are displayed in Figure 1. The retention times for the first-dimension liquid chromatography (^1^D-LC) and the second-dimension liquid chromatography (^2^D-LC) are represented horizontally and vertically, respectively. The compounds exhibited excellent separation, indicating the effectiveness of the method. Some of the peaks with larger peak volumes are labelled as P1–P39.

### 2.2. Identification of the Major Antioxidant Compounds by LC-MS/MS

The corresponding compounds of P1-P39 were fully or partially identified by referring to the standard substances and literature. These results are presented in Table 1. The results showed that the antioxidant components obtained through LC-ECD were mainly phenolic acids and flavonoids. The phenolic acids were predominantly derivatives of caffeic acid, such as monocaffeoylquinic acid (e.g., neochlorogenic acid, cryptochlorogenic acid, chlorogenic acid), di-caffeoylquinic acid (e.g., isochlorogenic acid A, isochlorogenic acid B, isochlorogenic acid C, and isomer P26) and tricaffeoylquinic acid (P20). In addition, there were also various isomers of lithospermic acid, namely polymers of caffeic acid, such as P14, P17, P26, and P29. The main flavonoids in the extracts were derivatives of luteolin, including luteolin’s C-glucoside such as orientin and isoorientin, as well as luteolin’s O-glucoside such as cynaroside and scutellarin. Other flavonoids detected were isoquercetin, casticin, and apigenin-7-glucoside. In addition, derivatives of *p*-hydroxybenzoic acid were also a major class of antioxidant components, such as P1, P2, P4, P32, and P37. Agnuside, a representative compound, was also a derivative of *p*-hydroxybenzoic acid and was the main active ingredient in *Vitex negundo* leaves that has been widely studied in the literature [32,33].

### 2.3. Antioxidant Activity Assays of Different Fractions Collected from ^1^D-LC

**Figure 2:**
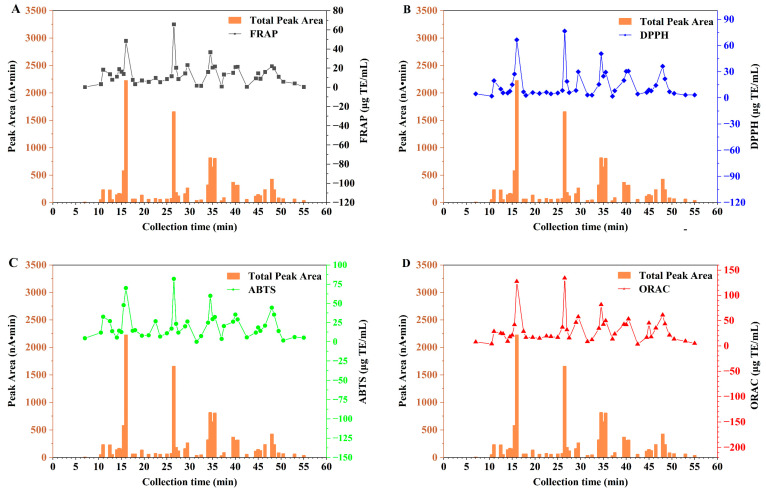
Peak areas and antioxidant activities of different fractions. (**A**) FRAP; (**B**) DPPH radical scavenging activity; (**C**) ABTS radical scavenging activity; and (**D**) ORAC.

Previous studies have shown that there was a significant linear correlation between the total peak areas of LC-ECD and total phenolic content of medicinal plant extracts [27,43]. Although LC-ECD can detect antioxidant compounds, it remains to be verified whether the antioxidant activity of individual compounds is correlated with its peak area. Therefore, the antioxidant activity of different fractions, namely the DPPH radical scavenging activity, ABTS radical scavenging activity, ferric reducing antioxidant power (FRAP), and oxygen radical absorbance capacity (ORAC) values were determined. As shown in Figure 2, the antioxidant activities of the fractions were varied in different time periods. The higher the total peak area of a fraction, the higher its FRAP value, ORAC value, DPPH radical scavenging activity, and ABTS radical scavenging activity. The fractions with greater antioxidant activity and their corresponding compounds were 16 min (chlorogenic acid), 26.5 min (isoorientin), 34–35.5 min (scutellarin, lithospermic acid isomer), 39.5–40.5 min (lithospermic acid isomer, cynaroside) and 48–48.5 min ((iso)orientin-hexoside).

Among the identified peaks, those with the largest volume were chlorogenic acid and isoorientin, suggesting that they may be the primary contributors to the antioxidant activity of *Vitex negundo* leaves. Chlorogenic acid has been previously reported to be a potential indicator for quality control in *Vitex negundo* [7]. Furthermore, chlorogenic acid and isoorientin have been demonstrated to possess robust antioxidant and anti-inflammatory properties, which can protect liver function, treat ulcerative colitis, and mitigate complications such as hyperglycemia and hyperlipidemia [43,44,45]. Consequently, our findings align with the existing literature and can be considered reliable and consistent.

### 2.4. Quantitative Analysis of Phenolic Compounds by LC-MS/MS

Quantitative analysis in multiple reaction monitoring (MRM) mode was performed based on the identification of compounds in *Vitex negundo* leaves. The parameters used for this analysis are shown in Table S1, which includes the higher response fragment ions.

#### 2.4.1. Method Validation

The linear equation was generated by plotting the peak area (y) against the concentration (x) of each compound. The limits of detection (LOD) and quantification (LOQ) of the compounds were calibrated using standard solutions with signal-to-noise ratios of 3 and 10, respectively. Intra-day and inter-day precision were analyzed by repeated injection of mixed standard solution six times on the same day and on three consecutive days, respectively. The standards were added to the samples of known concentration, which were then extracted and analyzed to calculate the recovery.

As shown in Table 2, the standard substances had good linear relationships with the correlation coefficient (R^2^) exceeding 0.99 within their linear range. The LOD of all antioxidants ranged from 0.07 to 5.23 ng/mL, and the LOQ ranged from 0.24 to 17.44 ng/mL. The RSD values of intra- and inter-day precision were all ≤ 7.87%. The average recovery of 19 compounds ranged from 90.01% to 102.31%, and the RSD values were ≤8.24%. The developed method showed good linear correlation, recovery, and precision.

#### 2.4.2. Sample Analysis

**Figure 3:**
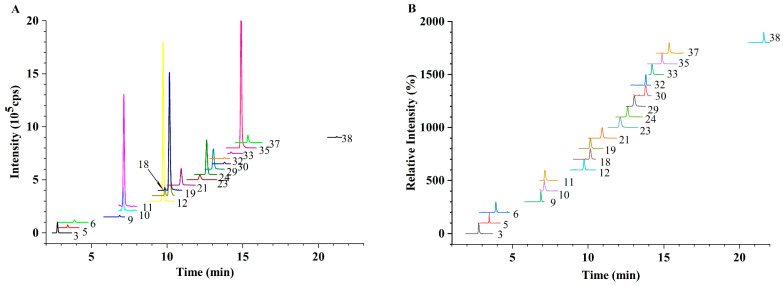
MRM chromatogram of standard solution (**A**) and *Vitex negundo* leaves (**B**).

Based on the proposed LC-MS/MS method, the contents of the 19 antioxidant compounds in 17 batches of *Vitex negundo* leaves were determined. The results are shown in Table 3 and the chromatograms of the standard solution, and the representative sample are shown in Figure 3A,B. The contents of the 19 compounds ranged from 0.15 to 33,025.99 μg/g, and the order of the average contents was as follows: agnuside > chlorogenic acid > isoorientin > ioschlorogenic acid B > scutellarin> ioschlorogenic acid A > isovitexin > casticin > cynaroside > ioschlorogenic acid C > protocatechuic acid > orientin > caffeic acid > apigenin-7-glucoside > cryptochlorogenic acid > neochlorogenic acid > isoquercitrin > vitexin > 3,4-dihydroxybenzaldehyde. It can be observed that the content of each component varies between different samples by approximately six to thirty times. This may be related to the location, time, and location of the collection.

Previous studies have demonstrated that the fractions identified by LC-ECD exhibit antioxidant activity. The peak area was positively correlated with the antioxidant activity of the fractions. In comparison with the content of these compounds, some components were found to have general antioxidant activity in vitro, but their content was particularly high in *Vitex negundo* leaves, e.g., agnuside and isovitexin. Although some components are not particularly abundant, their structural composition contains multiple phenolic hydroxyl groups, which enables them to exhibit a high response in LC-ECD and to contribute significantly to the antioxidant activity of *Vitex negundo* leaves, as exemplified by cynaroside.

### 2.5. Molecular Docking

**Figure 4:**
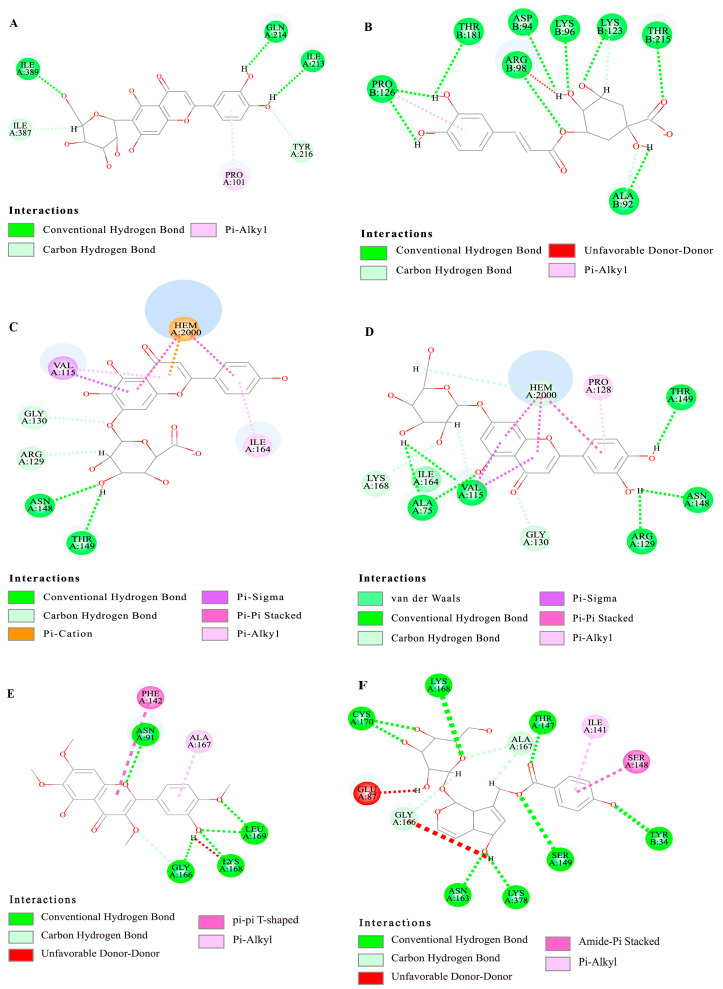
Molecular docking 2D diagram. (**A**) isoorientin—P4502C9; (**B**) chlorogenic acid—topoisomerase; (**C**) catalase—scutellarin; (**D**) catalase—cynaroside; (**E**) casticin—topoisomerase; and (**F**) agnuside—topoisomerase.

The main antioxidant substances screened from *Vitex negundo* were docked with antioxidant-related target proteins, respectively. The results are shown in Table 4. The Libdock score indicates the binding affinity of the small molecule with the protein receptor. The higher the Libdock score, the higher the degree of binding between small molecules and protein receptors. As shown in Table 4, isoorientin and chlorogenic acid interact with each antioxidant-related target protein. Cynaroside and scutellarin bind well with other enzymes except for superoxide dismutase and glutathione peroxidase. Isoorientin had a better binding degree than chlorogenic acid, with the highest binding to the P4502C9 protein. Isoorientin formed five hydrogen bonds with amino acid residues ILEA 389, ILEA 387, ILEA 213, GLNA 214, and TYRA216 on the target protein. The hydrophobic force was formed with PROA 101. Chlorogenic acid interacts best with topoisomerases, mainly forming 10 hydrogen bonds with PROB 126, THRB 121, ARGB 98, ASPD 94, LYSB 96, LYSB 123, THRB 215 and ALAB 92, and one hydrophobic force with PROB 126. Cynaroside and scutellarin have similar mechanisms of action on enzymes, and both have good interactions with catalase. They formed various interactions such as Van der Waals, conventional hydrogen bonds, carbon–hydrogen bonds, Pi-sigma, and Pi-Pi stacked, with amino acid residues in catalase (see Figure 4).

Agnuside and casticin are representative compounds in *Vitex negundo*, and it is known that agnuside has strong antioxidant and anti-inflammatory activities [35]. The results showed that agnuside had strong binding ability to each target protein, and the scores were all more than 100. It formed 11 hydrogen bonds with amino acid residues on topoisomerase, forming one Amide-Pi Stacked and Pi-AIkyl, while casticin had the worst score.

## 3. Materials and Methods

### 3.1. Reagents and Chemicals

Analytical standards of protocatechuic acid, chlorogenic acid, cynaroside, and caffeic acid were purchased from Aladdin Bio-Chem Technology Co., Ltd. (Shanghai, China). Orientin, isorientin, vitexin, isovitexin, ioschlorogenic acid B, ioschlorogenic acid A, ioschlorogenic acid C, agnuside, isoquercetin, and neochlorogenic acid were obtained from PufeiDe Biotech Co., Ltd. (Chengdu, China). Apigenin-7-glucoside and scutellarin were obtained from Sichuan Vikki Biotechnology Co., Ltd. (Chengdu, China). Casticin was obtained from Zhihua Pharmaceutical Co., Ltd. (Chengdu, China). 3,4-dihydroxybenzaldehyde was obtained from Beijing InnoChem Science & Technology Co., Ltd. (Beijing, China). The purity of all standards exceeded 97%. The standards were prepared as 1 mg/mL stock solutions in methanol, stored at −20 °C and diluted to the required concentration with 80% methanol before use. Methanol (HPLC grade), acetonitrile (HPLC and LC-MS grade), formic acid (HPLC grade), and ammonium formate (LC-MS grade) were purchased from Aladdin Bio-Chem Technology Co., Ltd. All remaining chemicals were of analytical grade. Ultrapure water was prepared using the Purelab Chorus II system (ELGA, High Wycombe, UK).

### 3.2. Plant Materials and Sample Preparation

Multiple batches of *Vitex negundo* leaves were collected from Qinzhou City, Guangxi Province in China, and were authenticated by Dr. Meng Lingjie (Zunyi Medical University, Zunyi, Guizhou Province, China). The fresh leaves were oven dried at 40 °C and powdered through a 50-mesh sieve.

The powdered samples (1 g) were extracted with 80% aqueous methanol (*v/v*) in an ultrasonic bath (40 kHz, 500 w) for 30 min. For the 2D-LC analysis, the volume of 80% methanol was 5 mL and for LC-MS/MS quantitative analysis, the volume was 100 mL. After centrifugation (9000 r/min, 5 min), the supernatant was filtered through a 0.22 μm nylon filter membrane.

### 3.3. Analysis of the Antioxidant Components in Vitex negundo Leaves by 2D-LC-ECD

#### 3.3.1. ^1^D-LC Conditions

The extract of *Vitex negundo* leaves was separated on a 2695e HPLC system (Waters, Milford, MA, USA) coupled with DAD and an Ultimate XB-C18 column (10 × 150 mm, 5 μm, Welch Materials, Inc., Shanghai, China) maintained at 35 °C. The mobile phase consisted of eluent A (0.05% aqueous formic acid (pH 2.7)) and eluent B (methanol) at a flow rate of 2.5 mL/min with the following gradient program: 0~10 min, 10~30% B; 10~40 min, 30~50% B; 40~50 min, 50~95% B; 50~52 min, 95~95% B; 52~53 min, 95~10% B; and 53~70 min, 10~10% B. The detection wavelength was 280 nm, and the injection volume was 50 μL. Fractions of the *Vitex negundo* leaves extract were collected every 0.5 min.

#### 3.3.2. ^2^D-LC Conditions

The fractions of *Vitex negundo* leaves obtained from ^1^D-LC were analyzed by an Ultimate 3000 Bio-RS system (Thermofisher, Waltham, MA, USA) coupled with ECD and a Phenyl-Hexyl column (3 mm × 150 mm, 5 μm) (Zhongpu, Fuzhou, China) maintained at 35 °C. The mobile phase consisted of eluent A (acetonitrile) and eluent B (50 mmol/L citrate solution, pH 2.75) at a flow rate of 0.5 mL/min with the gradient programs of Table 5. The detection potential was 700 mV, and the injection volume was 10 μL.

### 3.4. Identification and Quantification of Antioxidant Compounds by LC-MS/MS

The chemicals in *Vitex negundo* leaves were analyzed both qualitatively and quantitatively using on an I-class UPLC system coupled with a TQ-S triple quadrupole mass spectrometer (Waters, USA) with an electrospray ionization (ESI) source operating in the negative mode. The ESI-MS conditions were as follows: the capillary voltage of 3.5 kV was applied, the ion source temperature was set to 150 °C, the desolvent gas temperature was set to 500 °C, and the flow rate was set to 750 L/h. Fractions from ^1^D-LC were used for LC-MS/MS identification in Scan mode (*m*/*z* 100 to 900) and Product Ion Analysis mode. The mobile phase B was 10 mmol/L aqueous ammonium formate (pH 3.0), and the other conditions were the same as “Section 3.3.2”.

Sample solutions prepared at a liquid to material ratio of 1 g:100 mL were used for quantitative analysis of the compounds with MRM mode. The stationary phase was an XBridge BEH Shield RP18 column (2.1 mm × 100 mm, 1.7 μm, Water, USA), and the temperature was maintained at 45 °C. The mobile phase consisted of eluent A (acetonitrile) and eluent B (10 mmol/L aqueous ammonium formate, pH 3.0) at a flow rate of 0.4 mL/min, and the following gradient program was applied: 0–4.7 min, 5–7.5% A; 4.7–6.1 min, 7.5–12% A; 6.1–14.7 min, maintaining 12% A; 14.7–19.6 min, 12–19% A; 19.6–23.5 min, maintaining 19% A; 23.5–26 min, 19–45% A; and 26–27 min, 45–80% A. The injection volume was 1 μL.

### 3.5. In Vitro Antioxidant Assays of Different Fractions from ^1^D-LC

#### 3.5.1. DPPH Radical Scavenging Capacity Assay

The DPPH radical scavenging capacity was determined according to the method of Zhang et al. [46] with slight modification. The 100 μL of fraction was mixed with 100 μL of 0.125 µM DPPH solution and left in the dark at room temperature for 10 min. The absorbance of the mixture (A) was measured at 517 nm. The DPPH radical scavenging ability was calculated using the calibration curve (y = −0.0246x + 0.3127, R^2^ = 0.9994) of trolox, and the results were expressed as μg of trolox equivalents per mL of fraction (μg TE/mL).

#### 3.5.2. ABTS Radical Scavenging Capacity Assay

The ABTS scavenging capacity of the fractions was determined according to the reference of Al-Jaber [47]. Briefly, 100 μL of freshly diluted ABTS solution was mixed with 100 μL of the fraction and left in the dark at room temperature for 20 min. The absorbance of the mixture was measured at 734 nm. The ABTS scavenging capacity was calculated using the calibration curve (y = −0.0112x + 0.7014, R^2^ = 0.9948) of trolox, and the results were expressed as μg TE/mL.

#### 3.5.3. FRAP Assay

The FRAP value was determined according to the method described by Drakula et al. [48]. The 100 μL of the fraction was mixed with 100 μL of TPTZ reagent for 5 min, and the absorbance of the reaction mixture was measured at 593 nm as previously described. The FRAP value was calculated using the calibration curve (y = 0.0395x + 0.092, R^2^ = 0.9964) of trolox, and the results were expressed as μg TE/mL.

#### 3.5.4. ORAC Assay

The ORAC of fractions was measured according to the method modified by Tristán et al. [49]. A total of 25 μL of the fraction was mixed with 150 μL of 0.96 µM fluorescein in a 96-well black microplate and incubated at 37 °C. After 10 min, 25 μL of 119 mM 2,2′-azobis [2-methylpropionamidine] dihydrochloride was added, and the fluorescence intensity was measured every 5 min for 3 h with excitation and emission wavelengths of 485 and 538 nm, respectively. All solvents in this reaction were 75 mM phosphate buffer solution (pH 7.4). The ORAC was calculated using the calibration curve (y = 1.3964x + 8.6413, R^2^ = 0.992) of trolox, and the results were expressed as μg TE/mL.

### 3.6. Molecular Docking

Through references [50,51,52], target proteins related to antioxidant activity were screened. These included superoxide dismutase (PDB code: 2SOD), glutathione peroxidase (PDB code: 2HE3), catalase (PDB code: 1TGU), xanthine oxidase (PDB code: 3ETR and 3NRZ), topoisomerase (PDB code: 1ZXM), cytochrome P4502C9 (PDB code: 1OG5) and blood oxygenase (PDB code: 1N45). The target proteins were obtained from the RCSB website, and their three-dimensional structures were established. The main antioxidant compounds were selected as ligands and docked with different target proteins on DiscoveryStudio. The interactions between the compounds and proteins were then calculated.

## 4. Conclusions

In this study, we established an efficient and rapid method for screening and identification of antioxidants from *Vitex negundo* leaves by offline 2D-LC-ECD followed by LC-MS/MS. This method does not require complete separation of the sample, and compounds that respond well on 2D-LC-ECD have also been shown to have strong antioxidant activity in vitro. This demonstrates that LC-ECD is capable of rapidly and specifically identifying the majority of antioxidants, after which LC-MS/MS can be employed for the qualitative and quantitative characterization of the fractions. Finally, the molecular docking was employed to validate the principal components influencing the antioxidant activity of *Vitex negundo* leaves at the molecular level. Using a combination of the above techniques, it was demonstrated that the main components contributing to the antioxidant activity of *Vitex negundo* leaves were isoorientin, chlorogenic acid, agnuside, cynaroside, and scutellarin. This study also provides new avenues for research, methodologies, and technical support for the rapid screening of major antioxidant components from complex natural products.

## Figures and Tables

**Table 1:** Antioxidant compounds identified by LC-MS/MS in different fractions.

Peak	^1^D and ^2^D RT (min)	Precursor Ion (*m/z*)	Fragment Ion (*m/z*)	Identification	Standard Substances	Ref.
1	7.98, 3.20	299	137, 93, 101, 89	*p*-Hydroxybenzoyl-hexoside		-
2	10.15, 4.73	299	137, 89, 93, 101, 119	*p*-Hydroxybenzoyl-hexoside		-
3	10.26, 5.26	153	109, 91	Protocatechuic acid	Y	[5]
4	10.65, 4.14	311	191, 137, 93, 173	*p*-Hydroxybenzoylquinic acid		[5]
5	12.28, 5.15	137	109, 93	3,4-Dihydroxybenzaldehyde	Y	[5]
6	12.73, 5.05	353	191, 179, 161, 173, 135	Neochlorogenic acid	Y	[5]
7	14.53, 5.06	341	179, 161, 135, 177, 221, 149	Caffeoyl hexoside		[34]
8	14.07, 5.67	341	179, 135, 165, 177, 221, 149	Caffeoyl hexoside		[34]
9	15.24, 5.44	353	191, 179, 161, 173, 135	Cryptochlorogenic acid	Y	[5]
10	15.30, 5.66	179	135	Caffeic acid	Y	[5]
11	15.95, 5.97	353	191, 179, 161, 173, 135	Chlorogenic acid	Y	[5]
12	17.55, 4.71	467	167, 197, 235, 125, 287	Agnuside isomer	Y	[5,35]
13	19.41, 5.03	337	191, 163, 119, 173	*p*-Coumaroylquinic acid		[36]
14	21.10, 5,65	537	161, 423, 493, 323, 179, 151	Lithospermic acid isomer		[37]
15	22.54, 4.67	449	287, 151, 135	Tetrahydroxydihydroflavone-hexoside		[38]
16	22.37, 5.44	495	137, 151, 169, 125, 357, 213, 179, 281	Negundoside isomer		[35]
17	23.64, 6.05	537	161, 281, 519, 179	Lithospermic acid isomer		[37]
18	25.21, 4.14	447	327, 357, 297, 285, 339, 369	Orientin	Y	[39]
19	26.31, 4.95	447	327, 357, 297, 285, 339, 369, 429	Isoorientin	Y	[39]
20	27.55, 5.44	677	515, 353, 179, 335	Tricaffeoylquinic acid		[40]
21	29.38, 5.53	465	285, 137, 165, 303	Agnuside	Y	[5,35]
22	29.76, 5.33	431	311, 341, 283, 269, 353	Vitexin	Y	[5]
23	31.32, 3.60	495	281, 165, 137	Negundoside		[35]
24	32.56, 3.64	431	311, 341, 283, 269, 353	Isovitexin	Y	[5]
25	34.71, 3.92	461	285	Scutellarin	Y	[39]
26	35.88, 5.55	537	161, 493, 323, 151, 179	Lithospermic acid isomer		[37]
27	37.77, 4.76	515	191, 179, 353, 135, 161, 173	Dicaffeoylquinic acid		[37]
28	37.87, 4.28	449	287, 151, 135, 175	Tetrahydroxydihydroflavone-hexoside		[38]
29	39.54, 4.51	537	161, 439, 179, 123, 151	Lithospermic acid isomer		[37]
30	40.80, 4.37	447	285, 284	Cynaroside	Y	[5]
31	42.45, 3.24	463	301, 300	Isoquercitrin	Y	[41]
32	44.38, 3.96	473	173, 311, 137, 155	*p*-Hydroxybenzoyl-caffeoyl-quinic acid		-
33	45.41, 3.53	515	173, 179, 353, 191, 135	Ioschlorogenic acid B	Y	[5]
34	46.56, 3.84	515	179, 191, 353, 173, 135	Ioschlorogenic acid A	Y	[5]
35	48.09, 3.57	609	447, 429, 309, 327, 179	(Iso)orientin-hexoside		-
36	49.45, 3.33	431	268, 269, 311	Apigenin-7-glucoside	Y	[42]
37	50.56, 4.58	461	281, 179, 137	*p*-Hydroxybenzoyl-caffeoyl-hexoside		-
38	53.27, 4.03	515	179, 191, 353	Ioschlorogenic acid C	Y	[5]
39	55.19, 4.20	373	358, 343	Casticin	Y	[5]

**Table 2:** Method validation of the 19 compounds in *Vitex negundo* leaves determined by LC-MS/MS.

Peak	Compound	Linear Range	Calibration Equation	R^2 a^	LOD (ng/mL) ^b^	LOQ (ng/mL) ^b^	RSD (%) of Recovery ^c^	Recovery (%)	RSD (%) of Intra-Day (n = 6) ^c^	RSD (%) of Inter-Day (n = 6) ^c^
		(μg/mL)								
3	Protocatechuic acid	0.1~20	y = 8930.7x + 214.13	0.9990	1.75	5.80	6.84	90.04	4.44	5.13
5	3,4-Dihydroxybenzaldehyde	0.0015~0.3	y = 8283.9x − 225.96	0.9976	0.53	1.77	7.18	98.37	3.62	4.34
6	Neochlorogenic acid	0.025~5	y = 6842x − 40.4	0.9973	2.32	7.74	7.60	93.28	2.37	4.08
9	Cryptochlorogenic acid	0.005~1	y = 6882.7x + 30.76	0.9964	1.01	3.36	8.24	98.72	3.98	3.51
10	Caffeic acid	0.015~3	y = 20,659x + 779.8	0.9996	1.34	4.42	6.27	99.40	2.05	2.24
11	Chlorogenic acid	1.5~300	y = 9354.5x − 5320.1	0.9953	1.23	4.11	5.14	100.81	3.15	2.69
18	Orientin	0.05~10	y = 9393.7x − 933.43	0.9994	1.33	4.43	3.67	90.34	5.31	3.56
19	Isoorientin	1~200	y = 14,515x + 7915.9	0.9990	0.34	1.13	2.87	95.48	2.96	4.65
21	Agnuside	2~400	y = 10,103x + 4100.4	0.9999	0.59	1.96	1.29	99.15	2.56	4.17
22	Vitexin	0.005~1	y = 24,929x − 100.18	0.9992	0.44	1.40	6.08	90.01	4.34	3.05
24	Isovitexin	0.25~50	y = 22,757x + 853.01	0.9993	0.16	0.54	4.63	91.26	3.72	4.46
25	Scutellarin	0.5~100	y = 3957.7x − 72.5	0.9998	2.77	9.22	5.92	98.26	2.72	2.89
30	Cynaroside	0.25~50	y = 150,831x + 120.09	0.9933	0.44	1.45	3.15	102.31	5.82	3.26
31	Isoquercitrin	0.005~1	y = 2753.2x + 55.404	0.9968	0.07	0.24	6.45	98.01	7.87	3.50
33	Ioschlorogenic acid B	1~200	y = 5170.6x − 24.7	0.9983	1.41	4.70	7.71	96.72	4.70	5.03
34	Ioschlorogenic acid A	0.5~100	y = 762.71x − 30.502	0.9996	1.43	4.77	4.63	102.26	5.66	4.35
36	Apigenin-7-glucoside	0.025~5	y = 170,829x + 157.09	0.9944	0.68	2.28	3.48	99.46	6.69	2.09
38	Ioschlorogenic acid C	0.1~20	y = 4577.3x + 15.756	0.9994	0.54	1.80	6.05	99.35	4.23	4.48
39	Casticin	0.025~5	y = 449.64x − 89.347	0.9982	5.23	17.44	3.25	97.19	2.68	5.76

Note: (a): correlation factor; (b): limit of detection/limit of quantification; and (c): relative standard deviation.

**Table 3:** Contents of 19 antioxidant compounds in *Vitex negundo* leaves (μg/g).

Sample	Protocatechuic Acid	3,4-dihydroxybenzaldehyde	Neochlorogenic Acid	Cryptochlorogenic Acid	Caffeic Acid	Chlorogenic Acid	Orientin	Isoorientin	Agnuside	Vitexin	Isovitexin	Scutellarin	Cynaroside	Isoquercitrin	Ioschlorogenic Acid B	Ioschlorogenic Acid A	Apigenin-7-glucoside	Ioschlorogenic Acid C	Casticin
S1	133.82	1.58	14.13	25.85	48.74	4396.83	88.53	3450.61	31,196.04	15.29	1351.69	2966.35	571.18	2.01	1950.03	622.95	26.41	99.27	15.60
S2	280.23	0.83	20.35	44.06	52.53	13,565.91	116.93	4809.79	10,895.67	36.87	1758.09	4388.99	2199.23	27.64	8712.50	1500.21	133.00	575.01	33.74
S3	190.84	0.15	27.13	52.12	71.26	13,555.60	91.73	4799.64	7846.95	12.92	1788.45	5203.26	1727.71	59.42	12,588.38	1097.71	163.36	452.44	22.95
S4	165.97	1.14	44.91	38.26	27.87	12,383.11	27.96	3362.73	20,544.14	5.76	1028.52	2747.92	517.88	9.12	4461.85	3572.95	67.72	210.12	25.13
S5	160.44	0.35	10.37	42.64	33.74	5088.58	144.78	5749.69	15,453.19	32.04	1337.46	1264.42	723.87	3.69	1166.08	1236.44	17.35	250.19	19.02
S6	751.07	0.24	22.68	23.94	53.45	4855.33	17.15	4702.36	33,025.99	1.89	552.39	1165.94	355.27	8.20	367.41	1427.26	5.89	127.25	27.71
S7	177.55	2.48	30.82	53.01	28.69	5317.41	157.66	3299.59	14,968.56	17.74	675.36	1223.03	326.73	7.05	1060.54	1285.02	15.16	788.70	25.01
S8	196.21	2.10	6.56	19.81	50.18	3903.66	128.31	4070.43	8480.03	11.30	907.39	1802.83	203.00	4.91	503.96	937.73	7.07	122.21	8.31
S9	63.76	0.37	10.89	21.58	6.15	2770.59	13.22	651.32	21,366.24	2.62	379.93	667.47	101.74	1.67	414.89	258.95	6.41	50.50	9.35
S10	115.00	1.14	17.97	33.25	29.49	7864.67	168.04	4568.32	18,922.75	11.24	1006.33	1095.74	515.49	3.14	1591.28	1151.95	20.84	307.70	8.44
S11	27.58	0.81	12.94	1.54	8.11	4104.27	65.01	2706.27	4029.91	5.94	749.06	4125.97	778.72	6.96	3455.97	253.72	48.88	51.85	8.91
S12	25.12	0.71	15.02	7.83	30.31	8925.44	71.05	3333.65	28,064.92	6.13	648.31	2979.22	384.77	14.15	1008.46	428.02	15.36	51.24	6.95
S13	238.38	0.79	9.41	10.05	15.20	2677.15	13.38	2433.81	27,242.53	1.62	282.54	558.11	177.21	1.85	369.86	734.37	5.76	78.78	11.42
S14	46.74	0.20	13.10	32.40	39.11	12,020.38	36.42	3400.79	28,315.08	1.62	458.16	1365.22	323.89	10.67	685.49	1919.32	9.61	226.92	21.90
S15	260.24	2.07	7.35	40.84	11.21	1899.54	38.71	2607.33	11,343.23	3.25	466.33	1522.25	215.16	5.89	781.07	194.29	10.17	35.93	55.32
S16	209.41	1.36	5.12	19.02	5.39	1146.67	39.12	2793.99	9651.99	2.70	490.95	1032.92	256.58	5.40	912.03	198.98	14.84	33.15	58.53
S17	79.72	1.57	8.40	6.40	7.34	3407.66	21.12	2666.58	8132.60	2.11	572.99	2058.77	112.77	1.88	2049.63	1583.38	31.37	92.21	11.38
Average Value	183.65	1.05	16.30	27.80	30.52	6346.05	72.89	3494.52	17,616.46	10.06	850.23	2127.55	558.31	10.21	2475.26	1082.54	35.25	209.03	21.75

**Table 4:** LibDock score of six compounds.

Compound	Superoxide Dismutase	Blood Oxygenase	Cytochrome P4502c9	Catalase	Xanthine Oxidase	Glutathione Peroxidase	Topoisomerase
Isoorientin	116.57	93.75	120.25	116.43	109.37	86.48	116.41
Chlorogenic Acid	108.77	76.33	97.15	104.21	108.43	97.39	110.94
Cynaroside	-	97.90	160.22	187.57	135.80	-	76.65
Scutellarin	-	105.32	166.50	194.48	133.32	-	73.20
Agnuside	132.84	139.53	117.71	133.60	152.93	104.65	173.18
Casticin	46.26	74.79	44.62	65.64	70.34	71.06	105.06

Note: -: unable to dock.

**Table 5:** ^2^D-LC gradient programs.

^1^D-LC Fractions	0 min	8 min	11 min	11.5 min
7–17 min	8% A	20% A	80% A	80% A
17.5–22 min	10% A	30% A	80% A	80% A
22.5–30 min	12% A	35% A	80% A	80% A
30.5–41.5 min	20% A	40% A	80% A	80% A
42–57 min	25% A	50% A	80% A	80% A

## Data Availability

Data are contained within the article.

## Supplementary Materials

The following supporting information can be downloaded at: https://www.mdpi.com/article/10.3390/molecules29133133/s1, Table S1: MRM parameters of the 19 compounds.
