# Supplementary material for: Analysis of Antioxidant Compounds in Vitex negundo Leaves Using Offline 2D-LC-ECD and LC-MS/MS
Source: Molecules. 2024 Jul 1;29(13):3133. doi: 10.3390/molecules29133133 (PMC11242995; doi:10.3390/molecules29133133)
Supplement: Supplementary file 1 [file molecules-29-03133-s001.zip › molecules-3064019-SI.pdf]

Supplementary Material

Table S1. MRM parameters of the 19 compounds

Peak	Compounds	Precursor Ion (*m/z*)	Product Ions (*Quantifier/Qualifier, m/z*)	Cone Voltage （V）	Collision Energy （eV）
3	Protocatechuic acid	153.1	109/91	30	12, 15
5	3,4-Dihydroxybenzaldehyde	137.1	109/93	20	20, 22
6	Neochlorogenic acid	353.1	191/161	30	18, 20
9	Cryptochlorogenic acid	353.1	191/161	30	14, 20
10	Caffeic acid	179.1	135/107	38	16, 20
11	Chlorogenic acid	353.1	191/161	12	15, 20
12	Agnuside	465.1	285/137	25	20, 22
18	Orientin	447.1	327/357	40	25, 20
19	Isoorientin	447.1	327/357	30	22, 20
22	Vitexin	431.2	311/283	30	20, 30
24	Scutellarin	461.1	285/284	25	20, 20
25	Isovitexin	431.1	311/283	40	24, 28
30	Cynaroside	447.3	285/284	40	25, 25
31	Isoquercitrin	463.2	301/300	40	20, 20
33	Ioschlorogenic acid B	515.2	353.1/191	30	30, 35
34	Ioschlorogenic acid A	515.2	353.3/191	30	36, 35
36	Apigenin-7-glucoside	433.1	269/268	25	20, 20
38	Ioschlorogenic acid C	515.4	353.3/191	30	25, 30
39	Casticin	373.1	358/343	25	20, 22
